# Ultraviolet-Driven Deamination of Cytidine Ribonucleotides Under Planetary Conditions

**DOI:** 10.1089/ast.2019.2182

**Published:** 2020-07-08

**Authors:** Zoe R. Todd, Albert C. Fahrenbach, Sukrit Ranjan, Christopher J. Magnani, Jack W. Szostak, Dimitar D. Sasselov

**Affiliations:** ^1^ Department of Astronomy, Harvard-Smithsonian Center for Astrophysics, Cambridge, Massachusetts.; ^2^ Howard Hughes Medical Institute, Department of Molecular Biology and Center for Computational and Integrative Biology, Massachusetts General Hospital, Boston, Massachusetts.; ^3^ School of Chemistry, University of New South Wales, Sydney, Australia.; ^4^ SCOL Postdoctoral Fellow, Department of Earth, Atmospheric and Planetary Sciences, Massachusetts Institute of Technology, Cambridge, Massachusetts.

**Keywords:** Photochemistry, Pyrimidines, Nucleotides, Early Earth

## Abstract

A previously proposed synthesis of pyrimidine ribonucleotides makes use of ultraviolet (UV) light to convert β-d-ribocytidine-2′,3′-cyclic phosphate to β-d-ribouridine-2′,3′-cyclic phosphate, while simultaneously selectively degrading synthetic byproducts. Past studies of the photochemical reactions of pyrimidines have employed mercury arc lamps, characterized by narrowband emission centered at 254 nm, which is not representative of the UV environment of the early Earth. To further assess this process under more realistic circumstances, we investigated the wavelength dependence of the UV-driven conversion of β-d-ribocytidine-2′,3′-cyclic phosphate to β-d-ribouridine-2′,3′-cyclic phosphate. We used constraints provided by planetary environments to assess the implications for pyrimidine nucleotides on the early Earth. We found that the wavelengths of light (255–285 nm) that most efficiently drive the deamination of β-d-ribocytidine-2′,3′-cyclic phosphate to β-d-ribouridine-2′,3′-cyclic phosphate are accessible on planetary surfaces such as those of the Hadean-Archaean Earth for CO_2_-N_2_-dominated atmospheres. However, continued irradiation could eventually lead to low levels of ribocytidine in a low-temperature, highly irradiated environment, if production rates are slow.

## 1. Introduction

The elucidation of a potentially prebiotic synthetic pathway for activated pyrimidine ribonucleotides (Powner *et al.*, 2009) from simple starting materials provided one possible solution to a long-term issue with the RNA world: the synthesis of pyrimidine monomers. Recently, substantial progress has been made toward the potentially prebiotic synthesis of canonical and non-canonical RNA nucleosides (Becker *et al.*, 2018) and nucleotides (Powner *et al.*, 2009; Kim and Benner, 2017; Stairs *et al.*, 2017; Xu *et al.*, 2017; Becker *et al.*, 2019).

Powner *et al.* (2009) achieved the synthesis of β-d-ribocytidine-2′,3′-cyclic phosphate (denoted hereafter as C>p) from simple precursors, that is, glycolaldehyde, d-glyceraldehyde, cyanamide, cyanoacetylene, and phosphate. The irradiation of the mixture that forms C > p selectively degrades non-canonical ribonucleotide byproducts and affords partial conversion of C > p into β-d-ribouridine-2′,3′-cyclic phosphate (denoted as U>p). Later work also utilized partial conversion of C > P to U > p under ultraviolet (UV) irradiation, while additionally harnessing advantageous photoanomerization chemistry at an earlier synthetic step to increase the yield of the biological β-anomer via photoanomerization of α-2-thioribocytidine at a 76% efficiency (Xu *et al.*, 2017).

Powner *et al.* (2009) and Xu *et al.* (2017) used mercury arc lamps (primary emission at 254 nm) as a source of UV light, but such narrowband emission is not consistent with the spectral flux of the Sun on the surface of the early Earth. The lack of O_2_ and O_3_ in the atmosphere would have allowed mid-range UV light (200–300 nm) to penetrate to the surface of the planet (Cockell, 2000; Ranjan and Sasselov, 2017). This atmospheric scenario begs the question posed by Ranjan and Sasselov (2016): Would the UV photochemistry (here, specifically the partial conversion of C > p into U>p) actually occur at a realistic rate under the UV environment on the Hadean Earth? In this study, we address the photochemistry of C > p over a range of UV wavelengths and use these data to model the lifetime and concentrations of C > p under various environmental conditions.

The effect of UV irradiation on the pyrimidine nucleotides/nucleosides/nucleobases has been the focus of intense study, mostly due to an interest in DNA/RNA damage. In addition to the UV-induced formation of pyrimidine dimers, UV light can cause chemical changes to individual nucleobases, nucleosides, and nucleotides. Early work revealed that UV irradiation of uridine produces a hydrated species, namely 6-hydroxy-5,6-dihydrouridine (Wechter and Smith, 1968; Ducolomb *et al.*, 1976). This photo-generated hydrate can be converted back into uridine thermally or under highly acidic conditions (Sinsheimer and Hastings, 1949). Similarly, the product of UV irradiation of cytidine was postulated to be 6-hydroxy-5,6-dihydrocytidine based on hydrolysis to the uridine hydrate derivative (Johns *et al.*, 1965) and borohydride reduction (Miller and Cerutti, 1968). The irradiation product of cytidine was confirmed to be 6-hydroxy-5,6-dihydrocytidine by direct nuclear magnetic resonance (NMR) characterization (Liu and Yang, 1978).

**FIG. 1.**
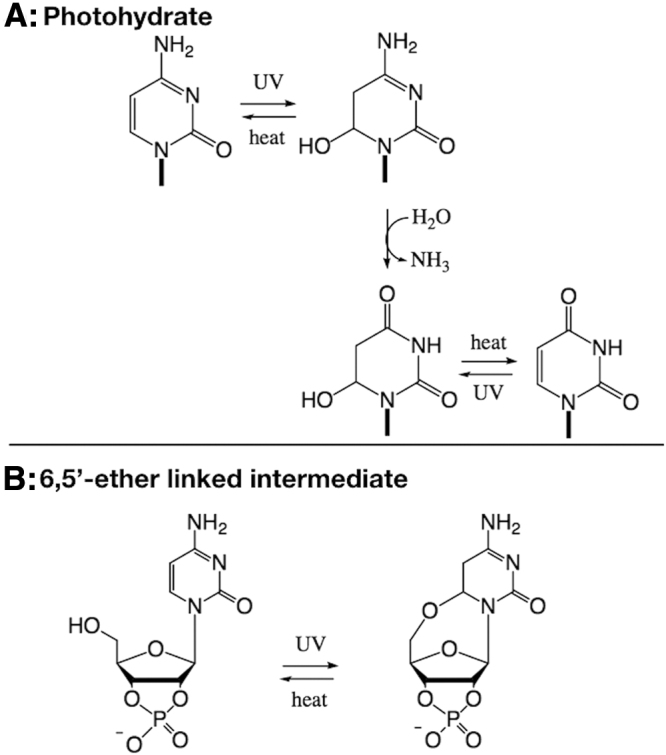
Two potential mechanisms for the photochemical processing of β-d-ribocytidine-2′,3′-cyclic phosphate. **(A)** Mechanism for cytidine, cytosine, and 5′-CMP, where UV irradiation produces the photohydrate (*e.g.*, 6-hydroxy-5,6-dihydrocytosine). This photohydrate can undergo deamination to the corresponding uridine photohydrate. Both photohydrates can be converted into the canonical nucleobase through thermal dehydration (the rate of thermal recovery of C is much greater than that of U). **(B)** Structure of the hypothesized intermediate on UV irradiation of β-d-ribocytidine-2′,3′-cyclic phosphate, suggested by Powner *et al.* (2009). Similarly, this intermediate could undergo deamination and then thermal recovery to β-d-ribouridine-2′,3′-cyclic phosphate. UV, ultraviolet.

On UV irradiation, the 270 nm band in the absorption spectrum of cytidine decreases, while a band at 240 nm appears (Sinsheimer, 1957; Wang, 1959; Wierzchowski and Shugar, 1961), resulting from absorption of the photohydrate, 6-hydroxy-5,6-dihydrocytidine (Fig. 1A). This photohydrate is unstable and can either revert to cytidine or, alternatively, deaminate to generate the uridine photohydrate (Schuster, 1964). The photohydrate of uridine, 6-hydroxy-5,6-dihydrouridine, then reverts to uridine, but on longer timescales than the cytidine photohydrate (DeBoer *et al.*, 1970).

Though cytosine, cytidine, and 5′-CMP form a photohydrated intermediate on UV irradiation, C > p was hypothesized by Powner *et al.* (2009) to form a different intermediate, in which the 5′-hydroxyl adds intramolecularly to C6 to give a bridged ether (Fig. 1B). Powner *et al.* (2009) hypothesized that elimination of the 5′-OH in the postulated photoproduct is a slower process in comparison to the dehydration of the analogous photohydrate, causing the C > p to undergo fewer cycles of photoexcitation, allowing some degree of protection from UV damage. Powner *et al.* (2009) observed that C > p is more stable to UV photodegradation than other cytosine nucleotides and nucleosides, including α-ribocytidine-5′-phosphate, β-arabinocytidine, β-arabinocytidine-5′-phosphate, and α-ribocytidine-2′,3′-cyclic phosphate, suggesting that UV may have played a role in selecting the canonical nucleotide stereochemistry due to increased stability. This is not the only suggestion of UV light playing a role in selection; in addition, the canonical nucleobases have shorter excited state lifetimes on UV irradiation than some other non-canonical bases (Beckstead *et al.*, 2016). Again, this promotes increased stability toward photoreactions of the canonical nucleobases, suggesting that UV potentially acted as a selection pressure on the early Earth (Lazcano *et al.*, 1988; Rios and Tor, 2013; Xu *et al.*, 2017).

In this study, we do not attempt to differentiate between the two possible intermediates, the photohydrate or the bridged 5′-6 cyclic molecule (Fig. 1), as the identity of the intermediate is not critical to the overall implications for the final products of the reaction and will not change our results. We focus on the UV-mediated partial conversion of β-d-ribocytidine-2′,3′-cyclic phosphate to β-d-ribouridine-2′, 3′-cyclic phosphate. In particular, we examine the UV wavelength dependence of this reaction in the context of the UV environment on the early Earth to assess the planetary implications for this reaction. We then use these data to model the concentrations and lifetimes of C > p for different environments on the early Earth.

## 2. Materials and Methods

To investigate the UV-driven conversion of C>p, a 50 μM solution of C > p in degassed, deionized water was prepared. Aliquots of this solution were irradiated from 215 to 295 nm individually in 10-nm internals with a 10-nm bandwidth. To perform the irradiation, a xenon arc lamp coupled with a diffraction grating acting as a monochromator to allow for tunable wavelength selection was used (as in Todd *et al*., 2018). The flux from the lamp over the wavelength interval 265–285 nm is within an order of magnitude of the surface flux expected over the same wavelength range on the surface of the early Earth (Ranjan and Sasselov, 2017).

Solutions were monitored by UV-Vis spectroscopy at 15-min intervals throughout irradiation, for a total duration of 2 h. On irradiation, the absorption band centered at 270 nm from C > p decreases and an absorption band at 240 nm grows in. These spectral changes were used to determine the concentrations of the starting material and photogenerated intermediate as a function of irradiation time, through experimentally determined extinction coefficients for both species (with concentrations determined by ^31^P-NMR, see Appendix A1, Figure A1). Once extinction coefficients were determined, the concentrations of the two species were determined from the UV absorption at the maximum wavelengths for the intermediate and starting material by solving a system of two equations, as described below:

$$A_{240nm}=\in_{C>p,240nm}c_{C>p}l+\in_{int,240nm}c_{int}l$$

$$A_{270nm}=\in_{C>p,270nm}c_{C>p}l+\in_{int,270nm}c_{int}l$$

We then determined the observed rate constant of the reaction by plotting the logarithm of concentration against time for each wavelength. Irradiations at each wavelength were performed in triplicate, to obtain an average rate constant and error. To compare reaction rates at different wavelengths, the observed rate constants were normalized by photon flux to get the rate constant, since the lamp used does not provide constant fluxes at all wavelengths. To do this normalization, powers at each wavelength were measured with a Newport power detector; they were then converted to a photon flux through the relation between wavelength and energy of a photon.

For experimental testing, the thermal reversion of the photoproduct of C>p, solutions of 50 μM C > p were irradiated in a Rayonet reactor (RPR-200, 254 nm, mercury lamps) for 15 min. Cuvettes were then placed in a Cary UV-Vis spectrometer with adjustable temperature control. Temperatures were varied from 25°C to 45°C, in 5° intervals. Each temperature tested was monitored for >16 h. UV-Vis absorption spectra were recorded and used to calculate concentrations to then determine reversion rates.

To study the partitioning of the photochemically generated intermediate between C > p and U>p, solutions of 50 μM β-ribocytidine-2′,3′-cyclic phosphate were irradiated for varying irradiation times (0, 10, 30, 60, 90, and 120 min) in the Rayonet reactor at 254 nm. After the set irradiation time elapsed, the samples were placed in a Cary UV-Vis spectrometer with an adjustable temperature control. Cuvettes were heated at 60°C for 100 h total, with UV-Vis monitoring every 30 min. The concentrations of C>p, U>p, and the photochemically generated intermediate were determined from solving a system of three equations from the absorption values at the maximum absorption wavelengths of the three species (240, 260, and 270 nm for the intermediate, U>p, and C>p, respectively):

$$A_{240nm}=\in_{C>p,240nm}c_{C>p}l+\in_{int,240nm}c_{int}l+\in_{U>p,240nm}c_{U>p}l$$

$$A_{260nm}=\in_{C>p,260nm}c_{C>p}l+\in_{int,260nm}c_{int}l+\in_{U>p,260nm}c_{U>p}l$$

$$A_{270nm}=\in_{C>p,270nm}c_{C>p}l+\in_{int,270nm}c_{int}l+\in_{U>p,270nm}c_{U>p}l$$

We note that the reaction likely contains another species: the uridine form of the intermediate. C > p photochemically generates the cytidine intermediate (either cytidine photohydrate or cytidine bridged ether intermediate, see Fig. 1). This structure then deaminates to give the uridine form of the intermediate. Thermal recovery generates U > p from any deaminated intermediate while regenerating C > p from any cytidine intermediate that did not deaminate. The uridine form of the intermediate does not absorb significantly in the 230–300 nm range (Appendix A2), so it should not interfere with the concentration determinations through UV absorption measurements.

## 3. Results

### 3.1. Wavelength-dependent UV conversion

**FIG. 2.**
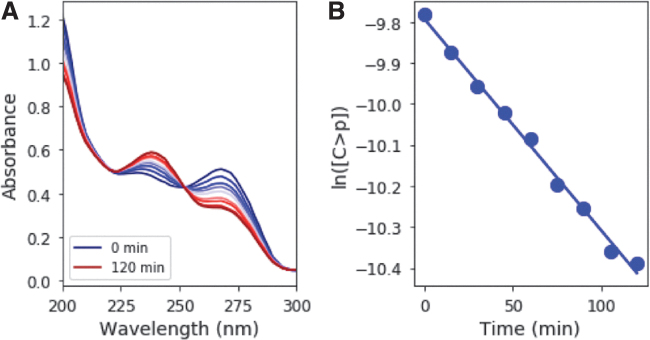
**(A)** UV absorption spectrum with irradiation at 265 nm in the tunable lamp setup. As irradiation is continued, the absorption at 240 nm increases whereas that at 270 nm decreases. This observation is due to the accumulation of intermediate and depletion of starting material, respectively. The concentrations of starting material and intermediate can be calculated for each time point from the UV-Vis absorption spectra. **(B)** Plot of logarithm of concentration of C > p with irradiation time. The slope of the best-fit line gives the observed rate constant.

**FIG. 3.**
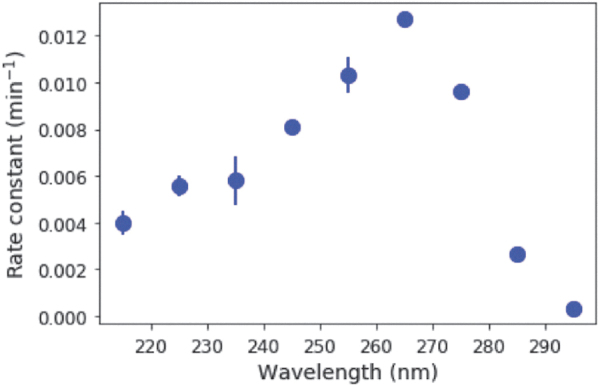
Rate constant of UV-driven reaction of C > p to the intermediate photoproduct as a function of irradiation wavelength, for a constant photon flux from 210 to 300 nm. The rate constant is greatest at a wavelength of 265 nm, as might be expected from the maximum absorption of C > p near this wavelength.

Irradiation of 50 μM C > p causes the initial absorption feature at 270 nm to decrease, whereas a band at 240 nm grows in (Fig. 2A). A clear isosbestic point is observed at ∼250 nm. This observation suggests that C > p is directly converted to the photoproduct, and only one photoproduct is formed initially. ^31^P-NMR spectroscopy supports this supposition by the fact that irradiation of C > p initially results only in one detectable ^31^P resonance other than the starting material. These spectral changes, coupled with experimentally determined extinction coefficients for the starting material and photoproduct (Appendix A1), can be used to determine their concentrations during irradiation. The slope of the best fit line for ln([C>p]) as a function of time gives the observed rate constant (Fig. 2B). Solutions were irradiated at 215–295 nm (10-nm interval, 10-nm bandwidth) in triplicate. Figure 3 shows the rate constant [for a constant photon flux of 5 × 10^14^ phot/s/cm^2^, expected from 210 to 300 nm based on the baseline early Earth scenario from (Ranjan and Sasselov, 2017), which uses atmospheric profiles from Rugheimer *et al.* (2015)] across a range of irradiation wavelengths, with the points representing the average of the triplicate set and the errors estimated by the standard deviation. The rate constant is maximum at 265 nm, which is consistent with expectations based on the absorption maximum of the starting material. The rate constant decreases significantly at wavelengths shorter than 245 nm and longer than 275 nm.

**FIG. 4.**
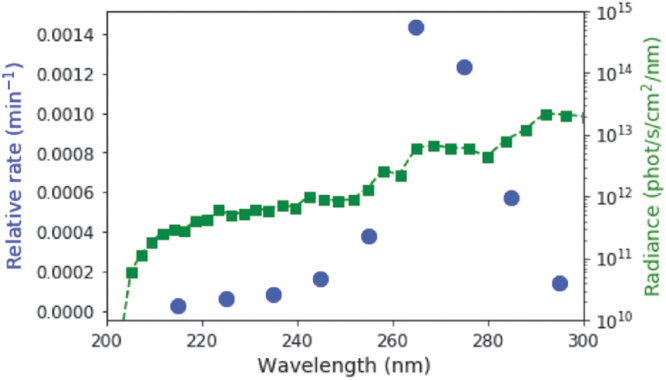
The relative rate of the conversion of C > p into the photochemically generated intermediate, taking into account the spectral surface flux (green squares) and the relative rate constants at varying wavelengths are shown in the blue points. The relative rate is maximum at 265 nm. The effective range of radiation for driving this reaction is from roughly 255–285 nm.

We next sought to compare the experimentally determined rate constants at various irradiation wavelengths with the spectral flux available on the surface of the early Earth from the Sun. A two-stream radiative transfer model produced and described in the work of Ranjan and Sasselov (2017) was used to calculate the surface spectral radiance through a sample N_2_/CO_2_-dominated (0.9 bar N_2_, 0.1 bar CO_2_) prebiotic atmosphere; the surface UV environment is robust to the uncertainties in the early Earth's atmospheric state as a result of saturation of absorption of wavelengths <204 nm due to CO_2_, resulting in mid-range UV wavelengths from ∼200 to 300 nm present on the surface of the planet (Ranjan and Sasselov, 2016, 2017). The intensities of longer wavelength UV light are greater than those of shorter wavelengths on the surface of the planet (see green line in Fig. 4). We then integrated the surface radiance by using the same 10-nm bins that the experiments employed, and calculated the relative rate of the reaction, defined as the product of the experimental rate and the integrated surface radiance (blue points in Fig. 4). The relative rate is still maximum at 265 nm, and the most productive wavelengths for driving this photochemical reaction in our model for the surface of the early Earth are 255–285 nm.

### 3.2. Thermal reversion

**FIG. 5.**
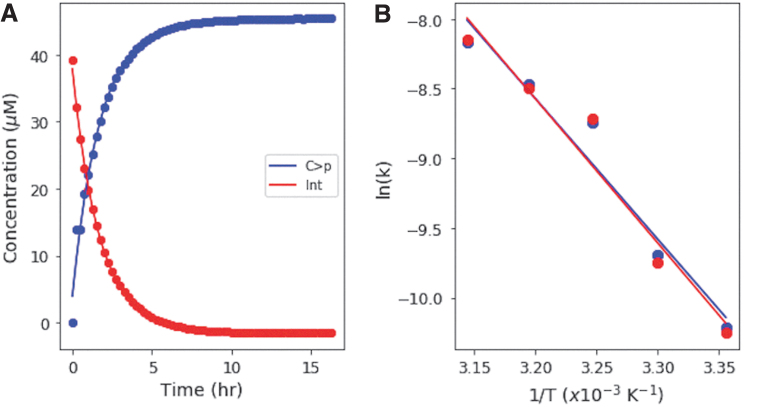
**(A)** Concentrations of C > p and the photogenerated intermediate during thermal recovery at 35°C. The concentrations are fit by exponential functions, which allow the rate constant of recovery to be calculated for a given temperature. **(B)** Arrhenius plot for both the rate of recovery of C and the rate of disappearance of the intermediate. The logarithm of the rate constant is plotted as a function of inverse temperature. The activation energies can be calculated from the slopes of the trendlines, giving activation energies of 84.0 ± 10.5 and 86.2 ± 11.7 kJ/mol for C > p and the intermediate, respectively, that is, the same within error.

On continued UV irradiation of C>p, the photochemically generated intermediate can either revert to C > p thermally, deaminate to form the uridine-derivative of the photochemically generated intermediate, or possibly undergo a second photochemical reaction. The photochemically generated uridine intermediate is more thermally stable than the cytidine photochemically generated intermediate (DeBoer *et al*., 1970), and hence the production of U > p requires prolonged heating. We investigated the thermal reversion of the UV-generated intermediate back to C > p at different temperatures to determine the activation energy for this reaction: 50 μM C > p solutions were irradiated in a RPR-200 Rayonet reactor (254 nm) for 15 min, until they were converted to at least 90% intermediate (as determined by UV-Vis spectroscopy). Then, solutions were incubated in the dark at temperatures from 25°C to 45°C, in 5° intervals for 16–24 h, while being monitored every 15–30 min by UV-Vis absorption spectroscopy. Concentrations of the starting material and UV-generated intermediate were determined and fit with exponentials as a function of incubation time. Figure 5A shows the concentration of the intermediate and starting material during heating at 35°C. The rates for thermal reversion to C > p were determined for various temperatures to generate an Arrhenius plot (Fig. 5B). The activation energy for this reaction was determined from both the appearance of C > p and the disappearance of the intermediate, as 84.0 ± 10.5 and 86.2 ± 11.7 kJ/mol, respectively, that is, the same within error. These were determined in the same reaction, with rates and activation energies calculated from the increase in C > p and the decrease in the intermediate, respectively. We did not see the appearance of U > p during the course of these experiments, since this requires elevated temperatures for longer periods of time.

### 3.3. Cytidine-derivative versus uridine-derivative partitioning

**FIG. 6.**
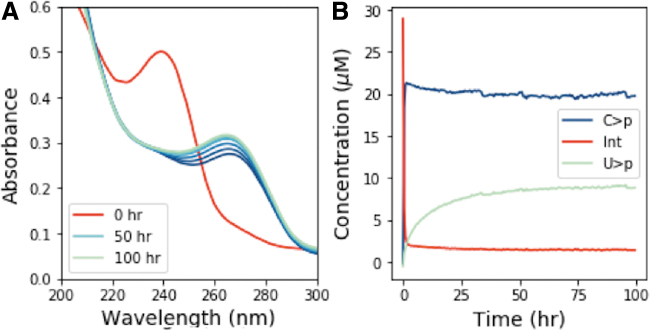
**(A)** Absorption spectra for various incubation times at 60°C after irradiation at 254 nm for 90 min. **(B)** Concentration of intermediate, C>p, and U > p as a function of incubation time, extracted from the absorption spectra. As heating is continued, the absorption spectra show increasing absorption at 260 nm, consistent with a thermal recovery of the deaminated intermediate to β-d-ribouridine-2′,3′-cyclic phosphate.

**FIG. 7.**
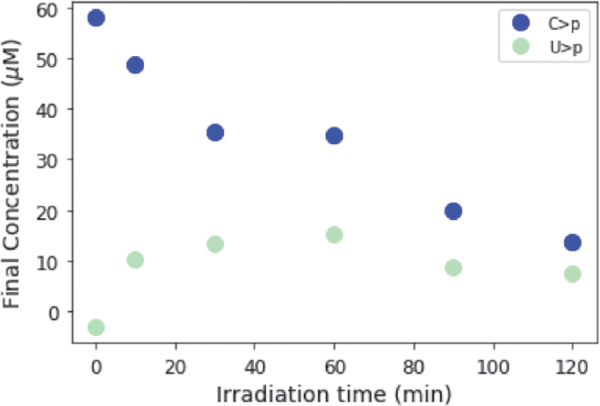
Final concentration of C > p and U > p after 100 h of incubation at 60°C, after irradiation for varying amounts of time (0, 10, 30, 60, 90, 120 min). During this longer heating step, the absorption spectra show increasing absorption at 260 nm, which indicates a recovery of the uridine intermediate to U>p. Longer irradiation times lead to an overall loss of material that is likely due to irreversible photodamage. At longer irradiation times, the ratios of C > p to U > p appear to plateau near 61% C > p and 33% U>p, after 100 h of incubation at 60°C.

On UV irradiation of C>p, the photochemically generated intermediate can deaminate to give the uridine form of the intermediate, which is more thermally stable to dehydration, and subsequent recovery to the canonical nucleotide than the cytidine intermediate. Accordingly, we next attempted to see the generation of U > p by irradiating C > p and then incubating the solution for a prolonged period at elevated temperatures. A solution of 50 μM C > p was irradiated for 90 min in the Rayonet RPR-200 reactor (254 nm); then, it was allowed to sit in the dark at 60°C for 100 h. Figure 6A shows the UV-Vis absorption spectra of this solution during the heating period after irradiation. The concentrations of C>p, the photochemically generated cytidine intermediate, and U > p were extracted from the UV-Vis spectra throughout the incubation and are shown in Fig. 6B. The intermediate is quickly returned to C>p, whereas it takes longer for U > p to appear, likely due to the uridine photochemically generated intermediate is more thermally stable. We do not attempt to quantify the uridine intermediate, as it does not show a clear absorption peak. It does not absorb significantly in the 230–300 nm window (Appendix A2), so the presence of this species in the reaction should not affect our overall determinations of the concentrations of the other species that are absorbed through the UV spectra. After 100 h at 60°C, the fractions of C > p and U > p reach a constant ratio. To better understand the partitioning between C > p and U > p and the role of irradiation, we allowed the initial irradiation time before incubation to vary. After irradiation (for 0, 10, 30, 60, 90, or 120 min), samples were incubated at 60°C for 100 h and monitored by UV-Vis. Figure 7 shows the final concentration of C > p and U > p after heating (100 h, 60°C) at various irradiation times. C > p is depleted more with increasing irradiation times, whereas the level of U > p after incubation seems to be approximately constant across different irradiation times. At the longer irradiation times, the proportion of C > p and U > p reaches a value of roughly 61% C and 33% U, though the total amount of material decreases with increasing irradiation times. This indicates that prolonged irradiation will deplete C>p, and it may also limit the amount of U > p generated, if production rates are slow.

### 3.4. Model day/night cycle

**FIG. 8.**
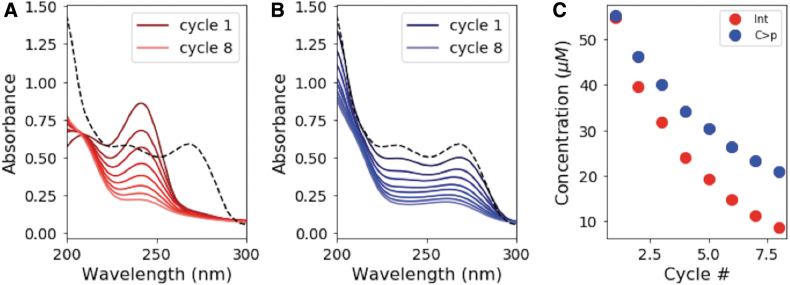
Degradation of material over repeated cycles of irradiation and thermal recovery. **(A)** Absorption spectra of the irradiated species (primarily intermediate) after each 15-min irradiation cycle. Each irradiation is followed by thermal recovery to make one full cycle. **(B)** Absorption spectra of the recovered (primarily starting) material as a function of cycle, after 24 h at 35°C. The black dotted line shows the absorption spectrum of the initial material (before irradiation). **(C)** Concentration of the intermediate and C > p after irradiation and thermal recovery, respectively, as a function of cycle number. With repeated cycles, material is lost; leaving roughly 88% of C > p recovered with each successive cycle.

We next studied what happens to C > p under repeated photocycling/thermocycling. We began with 50 μM C > p and alternated between irradiating in the Rayonet reactor (254 nm) for 15 min and incubating at 35°C for 24 h, repeating for a total of eight cycles. Figure 8A shows the absorption spectra for the solution immediately after each irradiation cycle, with the absorption spectrum of the starting material (before irradiation) shown by the dashed black line. Figure 8B shows the absorption spectra after the completion of the thermal step (24 h at 35°C after irradiation). With repeated cycles of irradiation and thermal recovery, material is lost, as seen by the decreasing absorption intensities. These spectra were converted to concentrations of C > p and the photochemically generated intermediate after each cycle (Fig. 8C). On average, only 88% of the material is recovered after a given cycle, leading to significant depletion of C > p by the end of the eighth cycle. The lost material is likely converted to the corresponding uridine intermediate or other irreversible photodamage. U > p is not observed under these experimental conditions, since generation of U > p from the uridine intermediate requires elevated temperatures for prolonged periods.

**FIG. 9.**
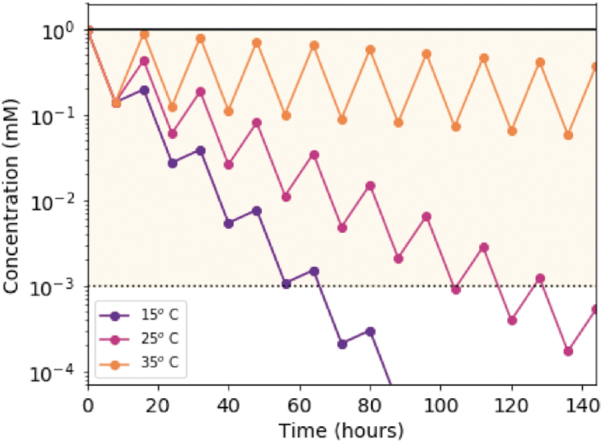
Model of the concentration of C > p through successive day/night cycles (16 h) from the experimentally determined wavelength- and temperature-dependent rates. The concentration of C > p is calculated as a function of time for temperatures of 15°C, 25°C, and 35°C. The irradiation degradation rate is determined by weighting the measured rate constants by the solar irradiation flux expected on the surface of the early Earth and integrating from 210 to 300 nm. We include degradation, by only allowing 88% of C > p to return with each successive cycle, leading to an overall loss with time. During these successive irradiation cycles, we would expect accumulation of the corresponding uridine intermediate, which could then be recovered as U > p on prolonged heating. The yellow shaded region indicates concentrations above 1 μM, which may be enough for prebiotic chemistry to occur.

This loss of material leads to a potential limitation of UV deamination of pyrimidine ribonucleotides. If a fixed stock of C > p undergoes repeated cycles of irradiation, it will ultimately be lost to U > p or other photoproducts, limiting the overall amount of C > p that could be available for prebiotic chemistry. This situation would be mitigated if the production of C > p occurs at a similar rate to the UV-induced loss. We asked what production rates of C > p are needed to sustain a steady state under a variety of planetary environmental conditions by simulating a day**–**night cycle expected on the early Earth (16 h total, *e.g.*, Lathe, 2006). Figure 9 shows the concentration of C > p (initially assumed to be 1 mM) under simulated day**–**night cycles. The net irradiation loss rate is calculated by weighting the experimentally determined rate constants by the solar irradiation flux calculated for the surface of the early Earth, and then integrating over 210–300 nm. The photochemical rate is assumed to be temperature independent, whereas the dark reaction is not. The thermal recovery during the dark is modeled at three temperatures: 15°C, 25°C, and 35°C. With each cycle, we impose a maximum recovery of 88% β-ribocytidine-2′,3′-cyclic phosphate, as indicated by our experimental results. This repeated photocycling depletes the initial stock of C > p on varying timescales depending on the temperature, where higher temperatures are more favorable for longer residence times of C>p. The yellow shaded region in Fig. 9 indicates levels of C > *p* > 1 μM, which is the threshold where prebiotic chemistry is believed to be plausible. C > p (initially at 1 mM) is expected to be >1 μM for 68, 130, and 1010 h for *T* = 15°C, 25°C, and 35°C, respectively. We find these results to be insensitive to the assumed length of the day.

This model can also be used to determine the production rates that are necessary to sustain a steady state of C > p at various concentrations, by equating the production and destruction rates. To maintain 0.1 mM C>p, production rates need to be from 0.3 to 4.4 μM/h, depending on temperature (Table 1). The required production rate scales linearly with steady-state concentration, for example, to obtain 1 mM C > p at steady state, production rates need to be 3.0–44 μM/h for the temperature range of 15–35°C. Past work on the prebiotic synthesis of molecules, including these nucleotides, has generally not focused on rates, but rather yields. These production rates offer a rough guideline as to what is required for the prebiotic chemistry to maintain a fairly constant stock of material without significant depletion under self-consistent conditions, and without invoking additional environmental constraints.

**Table 1.** Production Rate of C > p Necessary to Maintain a Steady-State Concentration of 0.1 mM, for Three Different Temperatures

Temperature (°C)	[C>p] = 0.1 mM steady state
	Production rate (μM/h)
15	4.4
25	2.3
35	0.3

Lower temperature environments require larger production rates. Production rates scale linearly with steady-state concentration, for example, a steady-state concentration of 1 mM C > p requires production rates of 23 μM/h at 25°C.

## 4. Discussion

Given that the spectral surface radiance on the early Earth is quite broad, and thus not well represented by narrowband irradiation sources typically used in irradiation experiments, it is important to analyze potential prebiotic photochemical reactions for their plausibility in the context of the UV environment on the early Earth. Previous studies showed a prebiotically plausible synthesis of β-d-ribocytidine-2′,3′-cyclic phosphate and subsequent UV-driven conversion to β-d-ribouridine-2′,3′-cyclic phosphate. The UV light (254 nm narrowband emission) also acted to destroy other synthetic co-products, enriching the relative concentration of the canonical ribonucleotides used by life today. To assess the prebiotic plausibility of the conversion of the C > p to U>p, we first studied the wavelength dependence of the reaction rate, as monitored by UV-Vis absorption spectroscopy. This analysis showed that the photochemical step proceeded best at an irradiation wavelength of 265 nm. In the context of the spectral flux available on the surface of the early Earth, generally, longer wavelengths are more accessible, meaning the reaction is more efficient under more realistic conditions. To quantify the effect of varying amounts of surface radiation at different wavelengths, we computed the weighted surface intensity, which accounts for the rate of the reaction as a function of wavelength and the intensity of the radiation available on the surface of the early Earth at each wavelength. Two hundred sixty-five nanometers are still the most efficient wavelength, with the window of usable radiation for this reaction occurring around 255–285 nm. Outside this window, either the reaction rate or the radiation intensity drops low enough that the weighted surface intensity suffers significantly. It is fortuitous that the most efficient wavelengths for this reaction are those that are not significantly screened out by plausible prebiotic atmospheric constituents and are available at sufficient intensity from the early Sun to drive the reaction. Our study demonstrates that the wavelengths that are necessary to drive this reaction would likely be available on the surface of the early Earth.

We further investigated the second step of the reaction, which is the thermal reversion of the photochemically generated intermediate back into the starting material. We determined the rate of the back reaction as a function of temperature and found the activation energy to be 85 ± 10 kJ/mol. On the prebiotic Earth, materials would be subjected to repeated cycles of light and dark during the day/night cycles. We modeled this by determining the net photochemical rate weighted over the wavelength intensities expected on the early Earth. After the irradiation step, we then modeled the dark reaction at various temperatures. Included in this model was the experimentally determined recovery of, on average, 88% of the starting material with each cycle. This ultimately leads to a limit on how long C > p would have been available on the early Earth, if C > p was only present in a finite amount. This calculation can conversely be used to constrain the necessary production rate of C > p to maintain steady-state concentrations. We find that a stock of 1 mM C > p will last from ∼70 to 1000 h at temperatures of 15–35°C, and that production rates of 3–44 μM/h are needed to maintain a 1 mM stock of C > p (depending on temperature). These considerations can help constrain the environmental conditions for the prebiotic chemistry to be self-consistent and plausible. In particular, if future experiments can quantify typical production rates under plausible planetary conditions, these can be compared to our findings to assess the overall consistency of the proposed chemistry. We also note that the deaminated product that forms as a result of UV light requires a significant amount of heating to return to U>p. This step would need to occur in the dark on the early Earth to not drive the reaction back to the UV-generated intermediate state. This condition again may imply constraints and requirements on the prebiotic environment if both C > p and U > p nucleotides are to be obtained through this UV-driven method. In particular, if both C > p and U > p are to be obtained through UV-driven deamination, the products would need to be incubated in the dark or exposed to significant durations of heating to recover the canonical ribonucleotides.

With these experimental constraints on this prebiotic chemical reaction, we can begin to envision an environmental scenario in which photochemistry can generate both cytidine and uridine nucleotides, but still have protection from overall UV degradation of both materials. If we invoke the geochemical scenario postulated by Patel *et al.* (2015), our analysis shows that the UV environment found on the surface of the early Earth in a shallow pond or lake would allow for conversion of β-d-ribocytidine-2′,3′-cyclic phosphate into β-d-ribouridine-2′,3′-cyclic phosphate on appropriate timescales. The production rates of C > p are currently not well constrained, but if production rates are non-negligible, the UV-driven conversion of C > p into U > p could lead to pools of both canonical pyrimidine ribonucleotides available on the prebiotic Earth. Even if production rates are slow, the material might be protected from further loss by UV light. Potential mechanisms for UV protection could include sinking deeper into a body of water, or being otherwise shielded by other UV absorbers (Sagan, 1973). We only examined the C > p to U > p photoreaction in isolation in this study; in actuality on the early Earth, other potentially UV-absorbing molecules may be present. These molecules could act to shield C > p and U > p from UV light, or could increase the rate of degradation through various photoredox or other chemical processes. These effects are beyond the scope of this study, but they are worth noting.

UV-driven photoconversion is not the only possible way to obtain both pyrimidine ribonucleotides on the early Earth. Reaction of cytidine derivatives with nitrous acid in the dark can generate uridine derivatives (Loring and Ploeser, 1948; Shapiro and Pohl, 1968). Recent work has suggested that nitrate and nitrite (collectively called NO_x_) may have been available in prebiotically relevant concentrations in shallow lake environments on the early Earth (Ranjan *et al.*, 2019). Thus, there are seemingly multiple plausible ways in which to convert cytidine nucleotides into uridine nucleotides on the early Earth. Possibly one or more could have been at play to provide both canonical ribonucleotides for the origins of life or the development of the genetic code. Multiple ways to achieve this conversion could make it easier for uridine production to be a larger-scale process, and less confined to a specific local geochemical environment.

## 5. Conclusions/Implications

Our study determined the prebiotic plausibility of the conversion of β-d-ribocytidine-2′,3′-cyclic phosphate into a photochemically generated intermediate that can partially convert to β-d-ribouridine-2′,3′-cyclic phosphate, in the context of the UV light available on the surface of the early Earth. Radiation at wavelengths from 255 to 285 nm is most efficient at driving this reaction; such irradiation was available on the early Earth. The relative proportion of C > p and U > p after UV irradiation appears to depend modestly on the length of irradiation time, with longer times reaching similar amounts of both. However, we find that continued irradiation (∼> 1 h in Rayonet RPR-200, 254 nm) leads to significant loss of the overall amount of C > p and U > p that can be recovered on heating. Proposed prebiotic chemistries for the accumulation and retention of C > p and U > p at, for example, 1 mM concentrations in the surficial environment must have production rates of several to tens of micromolar per hour to be plausible. If production rates are smaller, another mechanism for UV protection must be invoked to maintain these threshold concentrations for origins of life scenarios.

## Figures and Tables

**Figure:**
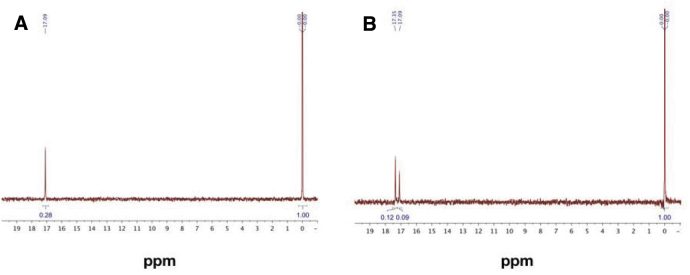
**FIG. A1.**  **(A)** ^31^P-NMR spectrum of initial 2 mM β-d-ribocytidine-2′,3′-cyclic phosphate, with TMP added as an internal standard. The starting material has a phosphorous signal at 17.09 ppm. **(B)** ^31^P-NMR spectrum of 2 mM β-d-ribocytidine-2′,3′-cyclic phosphate irradiated for 90 min in the Rayonet reactor at 254 nm. TMP was spiked in after irradiation as an internal standard. Two signals (17.09 and 17.35 ppm) are observed; the 17.09 ppm signal was confirmed to be the initial material by sample spiking. Integration of the two signals allowed for calculation of concentration and then extinction coefficients for the intermediate. NMR, nuclear magnetic resonance; TMP, trimethyl phosphate.

**Figure:**
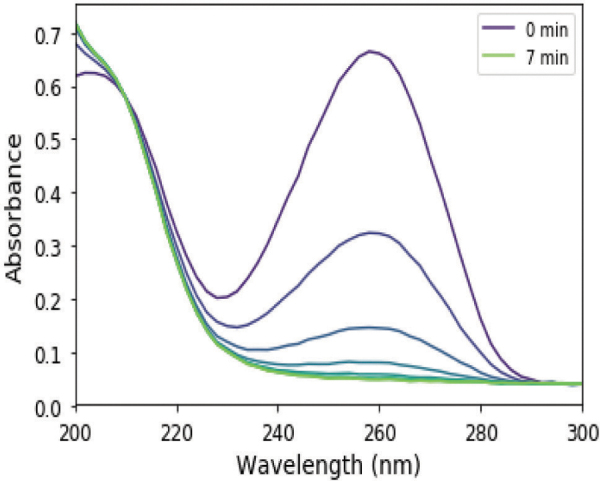
**FIG. A2.** UV spectra of U > p during irradiation (7 min total) in the Rayonet RPR-200 (254 nm). The uridine photogenerated intermediate does not absorb significantly in the 230–300 nm region. UV, ultraviolet.

## Abbreviations Used

NMR: nuclear magnetic resonance
TMP: trimethyl phosphate
UV: ultraviolet

## Appendix

### Appendix A1. Determination of Extinction Coefficients

To convert the observed absorbances into concentrations of both the starting material and the intermediate, the extinction coefficients for these species need to be known. We assumed that the extinction coefficients for the starting material (β-d-ribocytidine-2′,3′-cyclic phosphate) were the same for cytidine monophosphate (8814 and 9000 M^−1^ cm^−1^ for 240 and 270 nm, respectively). To determine the extinction coefficients for the intermediate, we obtained ^31^P-NMR spectra of a solution of 1 mM β-d-ribocytidine-2′,3′-cyclic phosphate before and after irradiation (254 nm, Rayonet reactor). The solution was monitored with ultraviolet (UV)-Vis absorption spectroscopy both before and after irradiation. An aliquot of the solution was spiked with a fixed concentration of trimethyl phosphate (TMP) as an internal standard to use for integration and concentration determination. The remaining unspiked solution was then irradiated in a Rayonet reactor (RPR-200, at 254 nm) and monitored by UV-Vis absorption until there was some conversion to the intermediate. We then spiked this sample with the same amount of TMP and took ^31^P-NMR spectra (Varian Inova 400 MHz spectrometer). The TMP peaks in the irradiated and unirradiated samples were used as an internal standard and to integrate the phosphorous signals from the sample. The unirradiated sample showed one peak at 17.09 ppm, whereas the irradiated sample showed two (17.09 and 17.35 ppm). Integrations of the signals were used to calculate the concentrations of the starting material and intermediate in the irradiated sample. We confirmed the identity of the starting material signal by sample spiking. With the concentrations of starting material and intermediate, as well as the absorption spectra, we could then solve for the extinction coefficients by solving the following set of equations for $\in_{int,240nm}$ and $\in_{int,270nm}$:

$$A_{240}=\in_{c,240nm}c_{c}l+\in_{int,240nm}c_{int}l$$

$$A_{270}=\in_{c,270nm}c_{c}l+\in_{int,270nm}c_{int}l$$

From this, we find that $\in_{int,240nm}=15,875M^{-1}cm^{-1}$ and $\in_{int,270nm}=2528M^{-1}cm^{-1}$.

### Appendix A2. Irradiation of U > p

To test the properties of the uridine form of the photochemically generated intermediate, we irradiated a solution of 50 μM U > p (254 nm, Rayonet reactor) over a span of 7 min. The UV absorption spectrum was recorded at various time intervals during the irradiation (Fig. A2). The initial U > p has an absorption maximum at 260 nm, but this feature quickly disappears with irradiation. By 7 min, almost no U > p remained. The photogenerated intermediate does not absorb significantly in the 230–300 nm range, which indicates that we can effectively ignore the contribution of this species to the absorption of solutions of irradiated and heated C>p. The only three species that should be detectable by UV spectroscopy are C>p, U>p, and the cytidine intermediate (absorption maxima at 270, 260, and 240 nm, respectively).

## Notes

Associate Editor: Nita Sahai
